# Pre-Launch Spectral Calibration of the Absorbed Aerosol Sensor

**DOI:** 10.3390/s23208590

**Published:** 2023-10-20

**Authors:** Jinghua Mao, Yongmei Wang, Entao Shi, Jinduo Wang

**Affiliations:** 1 Laboratory of Space Environment Exploration, National Space Science Center, Beijing 100190, China; maojinghua@nssc.ac.cn (J.M.); set@nssc.ac.cn (E.S.); 2 Beijing Key Laboratory of Space Environment Exploration, Beijing 100190, China; 3 Key Laboratory of Environmental Space Situation Awareness Technology, Beijing 100190, China; 4 School of Astronomy and Space Science, University of Chinese Academy of Sciences, Beijing 100049, China; 5 National Key Laboratory of Scattering and Radiation, Beijing 100854, China; jinduo_cas@163.com

**Keywords:** spectral calibration, spectral response function (SRF), absorbed aerosol sensors, slit homogenizer, slit function instrument

## Abstract

Spectral calibration consists of the calibration of wavelengths and the measurement of the instrument’s spectral response function (SRF). Unlike conventional slits, the absorbed aerosol sensors (AAS) are used as a slit homogenizer, in which the SRF is not a conventional Gaussian curve. To be more precise, the SRF is the convolution of the slit function of the spectrometer, the line spread function of the optical system, and the detector response function. The SRF of the slit homogenizer is a flat-topped multi-Gaussian function. Considering the convenience of fitting, a super-Gaussian function, which has a distribution similar to the flat-topped multi-Gaussian function, is employed to fit the measured data in a spectral calibration. According to the results, the SRF’s shapes resembling a Gaussian curve with a flat top could be derived, which contains a full width at half maximum (FWHM) of 1.78–1.82 nm for the AAS. The results show that the correlation is about 0.99, which indicates the usefulness of the fitting function that could better characterize the SRF of the instrument.

## 1. Introduction

### 1.1. Instrument Overview

Air pollution has always been an interesting topic worldwide [1,2,3]. China especially struggles with widespread and dense air pollution. Hazes cover most parts of the country, which has directed the focus on more comprehensive examinations since 2012. Most of the air pollutants are absorptive aerosols [4], mainly including dust storms, ash clouds, brown clouds, and haze. The primary purpose of the investigation of the AAS [5,6] is to obtain the optical thickness assessments of the aerosols and the absorbing aerosol index by detecting solar backscattering radiance in the ultraviolet and visible range.

**Figure 1:**
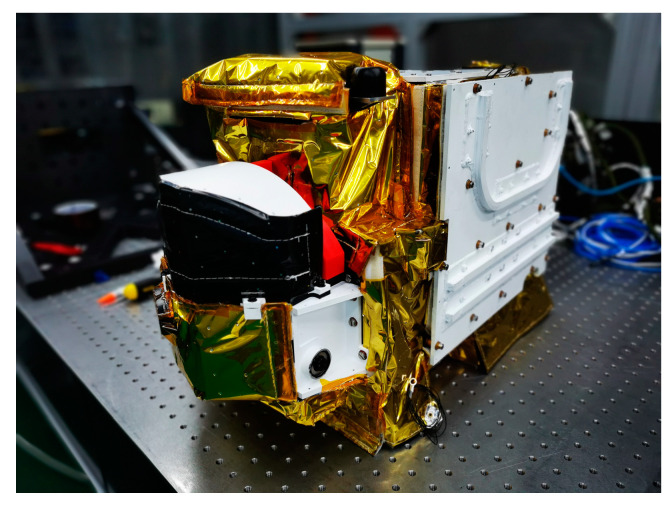
A depiction of the AAS.

The AAS is one of the payloads aboard the GaoFen-5B satellite. A UV-VIS spectrograph integrates both high spatial resolution and low spectral resolution to generate global coverage daily. The origin of air pollution can be analyzed using high-ground pixel projection and considerable temporal resolution. Hence, the AAS has been employed as the first tool to obtain aerosol observations by gauging a continuous spectrum interval of 340–550 nm in China. A 114° field-of-view (FOV) and a 4 × 4 km^2^ spatial resolution at Nadir are used, which is the highest spatial resolution among similar payloads. The GF-5B satellite, launched at 10:30 UTC on 6 September 2021, is a sun-synchronous satellite with an orbit height of 705 km, an orbit period of approximately 101 min, and has a primary mission to conduct chemical analyses of the atmosphere, including aerosol and high-resolution land observations. This paper presents the AAS instrumental descriptions and initial observations during the first year in the orbit. The main specifications are summarized in Table 1. Generally, the AAS has a wide FOV, with a pushbroom imaging spectrograph using a CCD detector to realize spectral and spatial imaging processes. The telescope, with two freeform mirrors and a 3-D slit, can achieve the critical spatial requirement. Except for the Nadir mode, the AAS contains a Sun port for in-orbit calibration, employing two diffusers. The diffusers enable the illumination of the spectrometer’s entrance slit whether irradiation is led by the sun or an internal white light source. The calibration is carried out at all wavelengths and a total FOV of 114°. A photograph of the AAS is shown in Figure 1.

The AAS’s main specifications are summarized in Table 1.

This article includes four sections. Following the introduction and the instrument description in Section 1, the calibration equipment and procedure are introduced in Section 2. Section 3 presents the results of spectral calibration, which have a good characterization of the instrument. The conclusion is presented in Section 4.

### 1.2. The Spectral Calibration

The wavelength calibration [7,8] and confirmation of the spectral response function (SRF) [9,10,11] are the components of spectral calibration. To establish a characteristic wavelength for each detector pixel, the wavelength calibration also yields a wavelength map *λ*(*x_ij_*), where *x_ij_* represents a detector pixel. The characteristic wavelength of a pixel is defined as the wavelength for which the response of the central peak of the instrument resides at the center of the specified pixel.

The monochromatic image at the entrance slit of the spectrometer, located upon the detector, is represented by the slit function [12]. The former wavelength is known as the source wavelength, for which the central peak of the instrument response is located at a specified pixel. The latter describes the spectral response of each pixel at varying wavelengths. The SRF is modeled by a function *y*(d*λ*), in which *y* denotes a detector pixel and d*λ* represents the difference between the pixel characteristic wavelength and reference wavelength in nanometers (nm). Concerning all the wavelengths and accurate knowledge of the SRF, the FOV is crucial for the products and spectral calibration.

**Figure 2:**
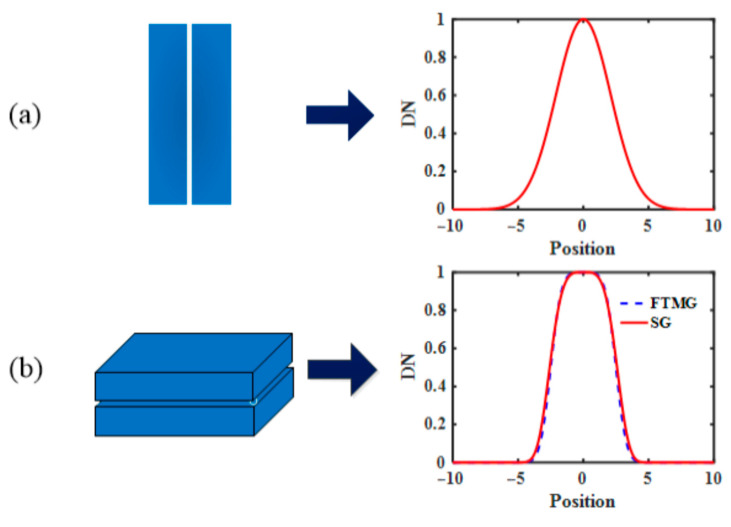
(**a**) The conventional slit and its SRF, and (**b**) the slit homogenizer and SRF of the AAS. FTMG, flat-topped multi-Gaussian; SG: super-Gaussian.

Different from conventional slits, the AAS employs a slit homogenizer [13,14,15,16], which has an SRF that does not represent a conventional Gaussian curve [12,13], as shown in Figure 2. The SRF of the slit homogenizer is a flat-topped multi-Gaussian function. Considering the convenience of fitting, a super-Gaussian function, which has a distribution similar to the flat-topped multi-Gaussian function, was utilized to fit the measured data in a spectral calibration.

Figure 2 depicts the SRFs relative to different types of slits. The application of the slit homogenizer could make the spectral direction imaging (along the orbit direction) more uniform, which means that different wavelengths approach the same shape and full width at half maximum (FWHM). To accurately depict the slit function of different wavelengths, a dedicated slit function instrument (SFI) was developed [12]. The SFI comprises an echelle grating monochromator, which can simultaneously produce several spectral lines at small intervals. Using a 300 W xenon arc lamp (Hamamatsu super quiet) as a light source, the slit function measuring instrument adopts the splitting mode of an echelle grating light. Through the secondary scattering by the spectrometer, the evenly distributed emergent light forms a multiline on the image surface and can advantageously obtain the SRF of multiple spectral lines in the working band of the instrument simultaneously, which improves the testing accuracy and reduces the operational complexity.

In this paper, the spectral calibrations of the AAS were carried out before the launch. The measurements were taken over the 340–550 nm spectral range with an FOV of 114°. The calibration was performed in a thermal vacuum environment, where the wavelength shift and the temperature-dependent SRF were analyzed. The standard spectral line lamps used for the calibration was produced by the The National Institute of Metrology, China(NIM), the SFI was self-developed, and the software was the AAS Ground Calibration Processing Software (GCPS), version 1.1.

## 2. Methods

### 2.1. .Equipment for Calibration

Spectral calibration deals with two main factors, which are called the slit function instrument (SFI) and the wavelength registration. The wavelength registration is performed in the Sun port, while the SRF is measured in the Earth port. To ensure the validity of the calibration data, the AAS is placed in the vacuum chamber during the calibration. The vacuum level of the system is represented by 6 × 10^−4^ Pa, and the temperature of each part of the AAS is controlled by the operating temperature of the satellite.

A spectral line source prompts a limited quantity of spectral characteristics with well-known wavelengths and narrow spectral bandwidths. So, it is imperative to choose the appropriate spectral line source. Therefore, four kinds of spectral line sources were employed, which are mercury (Hg)- [17], argon (Ar)- [18], krypton (Kr)- [19], and Neon (Ne)- [20] type lamps.

To enable an accurate measurement of the SRF, an optical source was designed that employs an echelle grating to generate an output beam that contains around 30 spectral peaks within the wavelength range of 340–550 nm, which relates to a substantial amount of diffraction orders for the echelle grating according to the grating diffraction equation presented in Equation (1).

(1) sinα+sinβ=mλ/d

where α denotes the angle of incidence of light, β represents the diffraction angle, *m* denotes the diffraction level, *λ* represents the center wavelength, and *d* denotes the grating constant.

The dispersion of the grating inversion is derived in Equation (2).

(2) $$\frac{d\lambda }{dl}=\frac{10^{6}\cos\beta }{mnf}$$

where *n* represents the grating inscription density, *dl* denotes the exit slit width, and *f* represents the exit focal length. Then, Equation (2) can also be represented by Equation (3).

(3) $$d\lambda =\frac{10^{6}\cos\beta }{mnf}dl$$

Equation (3) represents the spectral broadening corresponding to the slit width, i.e., the spectral resolution corresponding to different wavelengths.

The echelle grating used in the SFI was inscribed at 79.01 grooves/mm, and the diffraction angle was set to 71.5°. According to Equation (2), when the focal length of the collimator lens, *f*, is assigned to 615.894 mm, the spectral resolution of the measurement instrument of the slit function ranges between 0.03 nm and 0.06 nm for the spectral range of 340 nm–550 nm; the linewidth was set to 3% of the spectral resolution of the AAS, which is sufficient for the calibration of the slit function.

The absolute wavelength for the SFI lines was determined by the use of a wavelength calibration of the AAS. On the rotation of the echelle grating, the wavelengths of the lines that are produced may be shifted stepwise by 0.05 nm. The detector pixel’s response to an echelle peak produces the spectral slit function for the relevant pixel. The rotation of the echelle grating provides the shape of the spectral slit function. This is measured with a larger sampling and higher accuracy when compared to those determined using a spectral line source, for which the accuracy is limited by the pixel sampling of the detector’s spectral resolution. Because of the extensive FOV of the AAS, the calibration was performed on a rotating platform, and the results were used to calculate the spectral smile of the AAS. The stimulus properties for the wavelength calibration and SFI tests are shown in Table 2 and Table 3, respectively.

### 2.2. Calibration Setup

**Figure 3:**
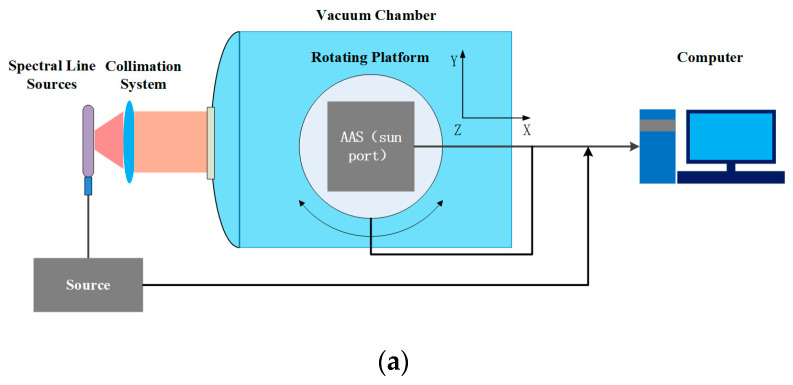

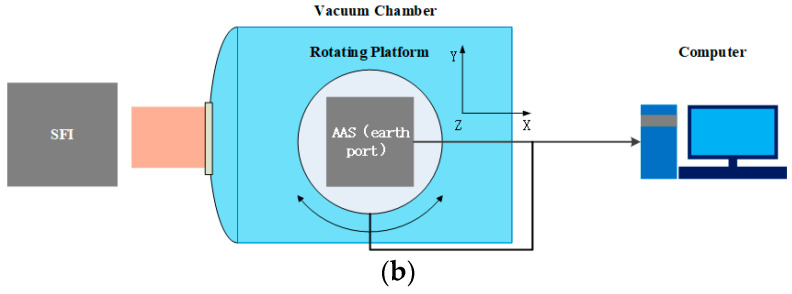
(**a**) A schematic diagram of the experimental setup for the AAS wavelength calibration. (**b**) A schematic diagram of the experimental setup for the AAS-SFI calibration.

**Figure 4:**
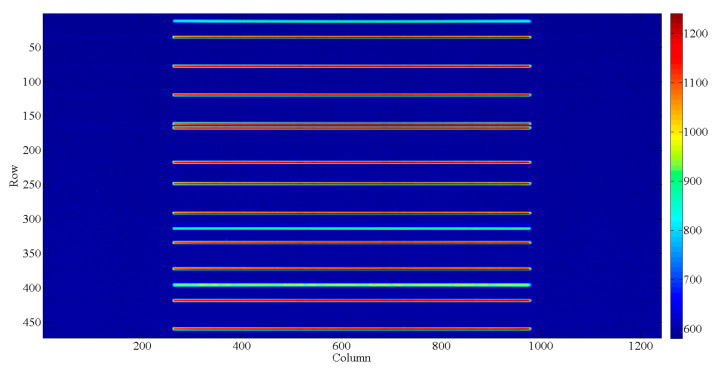
The spectral distribution acquired by the CCD during wavelength calibration. The different color bars represent different DNs.

The AAS calibration occurs in a vacuum chamber, which has a better vacuum than 6.4 × 10^−3^ Pa, with a cleanliness of 1000. The AAS has a thermal control system ensuring that the temperature is consistent with the AAS in orbit. Because the spectral smile does not differ with wavelength, it is necessary to calibrate all pixels of the AAS in the FOV to obtain the wavelength map. However, the wavelength calibration measurements of the Sun port have a relatively lower SNR. Thus, the wavelength calibration is unworkable when offsetting for the deficiency. Therefore, a special collimating system was designed for the spectral line source that improves the luminous flux. The lenses for the collimation system were purchased from Edmund and the information on the light source after the collimation is shown in Table 2. The AAS solar port has a diffuser, which shares one optical system with the Earth port. Each calibration can only cover 2–3 pixels in the spatial dimension and needs to be rotated many times with the rotary table to cover all the spatial dimensions of the CCD pixels. On the other hand, light from the solar port passes through the diffuser and can be calibrated to cover the whole FOV of the AAS at one time, and in this way, the full image-plane spectral maps of the CCD in the calibration are attained. The experimental setup is shown in Figure 3. Multiple spectral lines of the spectral sources are detected by the AAS, as shown in Figure 4. The known spectrum suggests that the wavelength of the other pixels can be obtained by interpolation or the least squares fitting method.

The SRF calibration is performed from the Earth port. A depiction of the SRF calibration is shown in Figure 3b. The AAS uses spectral line sources to obtain the operating wavelength of each pixel. The SFI is utilized to determine the slit function of the AAS. The peaks formed by the SFI on the AAS are then used to obtain the center wavelength based on the calibrated AAS, and a small adjustment of the angle of the middle-stepped grating is implemented to attain the SRF of the AAS. The SRF is obtained by employing two different methods: one is the AAS response to a single narrow wavelength that relates to a single echelle angle measurement, and the other is via the rotation of the echelle grating, in which the wavelengths of the lines that are produced can change by a step size of 0.1 nm. The sampling frequency is a spectral resolution corresponding to 4 pixels of the AAS, and the pixel size of the CCD is 22.5 μm × 22.5 μm. Therefore, the SRF with low sampling points could be obtained by utilizing the first method. The rotation of the echelle grating provides the shape of the spectral slit function. The latter is measured by employing more sampling and achieves higher accuracy when compared to utilizing a single echelle angle, where the accuracy is limited due to the pixel sampling of the spectral resolution via the detector. Given a field, the SRF for each pixel can be obtained by fitting these sampling points with a super-Gaussian curve and the FWHM of the SRF represents the spectral resolution of the AAS. This enables the spectral smile of a specific wavelength to be attained.

## 3. Results and Discussion

### 3.1. Wavelength Calibration

**Figure 5:**
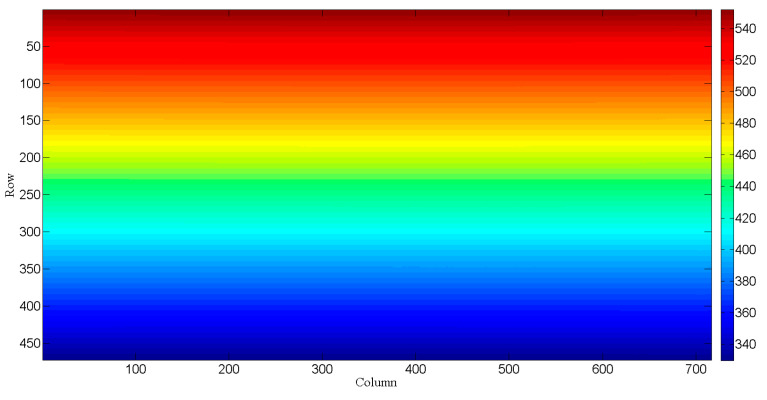
The spectral distribution on the CCD by wavelength calibration. Different color bars represent different spectra.

**Figure 6:**
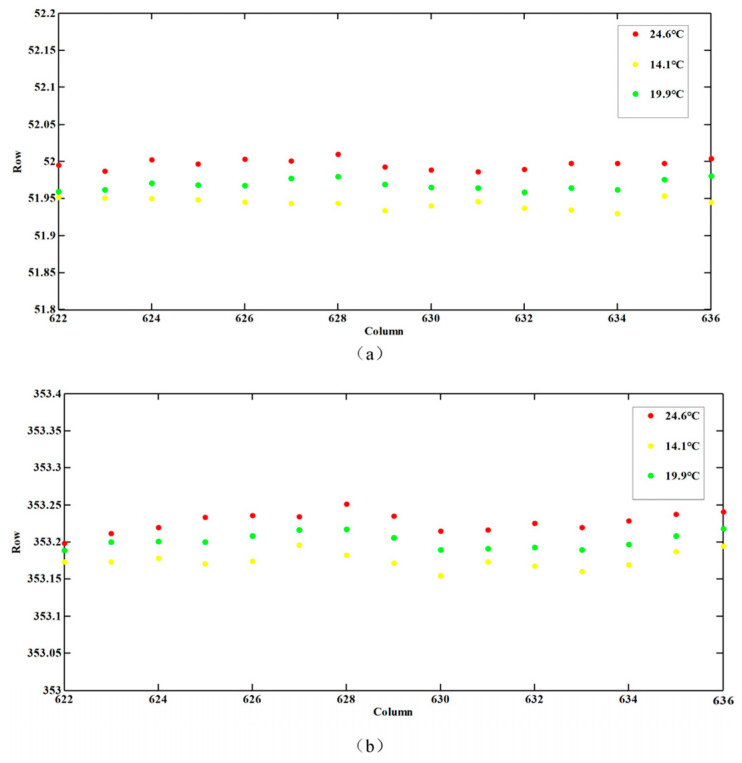
The wavelength shifts on the CCD at different temperatures for the wavelengths of (**a**) 546 nm and (**b**) 404 nm.

Measurements employing three spectral line sources deliver precise information on wavelengths. The relationship between wavelengths and pixels can be established by a cubic spline interpolation. Light travels in different media with constant frequency, *f*. If the wavelength in a vacuum is denoted by *λ*_0_, and the wavelength in a medium with a refractive index *n* is represented by *λ*, then C =*λ*_0_ × f can be written where *f* denotes the frequency and V = *λ* × *f,* where V represents the speed of light in the medium; *n_λ_* = C/V, where C represents the speed of light in a vacuum, which gives *λ* = *λ_0_*/*n_λ_*_,_ where *n_λ_* denotes the refractive index. Based on the expression of the air refractive index, the refractive index at different wavelengths is calculated, and finally, the wavelength is corrected according to the deviation. The results of the wavelength calibration are shown in Figure 5. The spectral range determined from the wavelength calibration ranged from 338 to 551 nm for the AAS detector, and the dispersions were 0.47 nm/pixel. The wavelength calibration accuracy was 0.03 nm. To better understand the optical properties of the AAS, the temperature dependency of the wavelength shift was examined during the on-ground calibration operation. The position change of the characteristic wavelength was obtained at three different temperatures, including an extremely high temperature of 24.6 °C, an extremely low temperature of 14.1 °C, and a working temperature of 19.9 °C, respectively. The results are shown in Figure 6. It was found that the maximum wavelength shifts of the two extreme temperature conditions were less than 0.06 pixels, which could be corrected in orbit by using the Fraunhofer structures within the solar spectrum [21].

### 3.2. SRF Calibration

**Figure 7:**
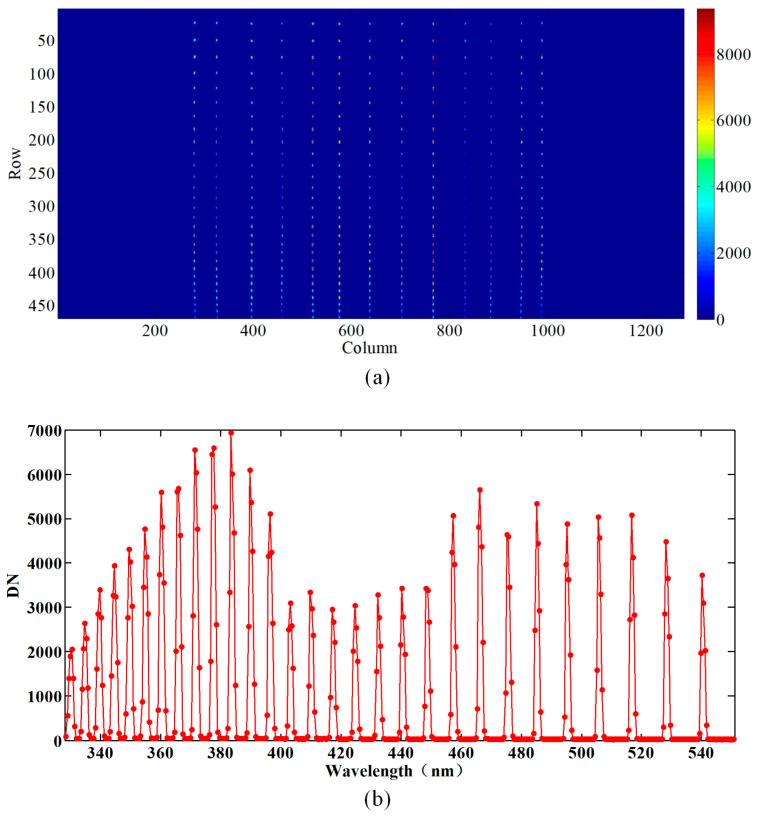
(**a**) The map of the slit function from different fields using the SFI at different angles, in which rows and columns denote the spectral and spatial dimensions, respectively; the different color bars represent different DNs. (**b**) The distribution of the spectrum obtained by extracting the center FOV.

Optical equipment has been designed to accurately measure the SRF. The equipment employs an echelle grating to produce a 50 mm beam, which contains a tunable, uniform separation, and narrow spectral peaks in the range of 340–550 nm. Here, a slit function of 114° was tested by the rotating platform with a step size of 10°, while a spectrum of the Nadir observation was obtained by an extraction in the middle of the map, as shown in Figure 7.

**Figure 8:**
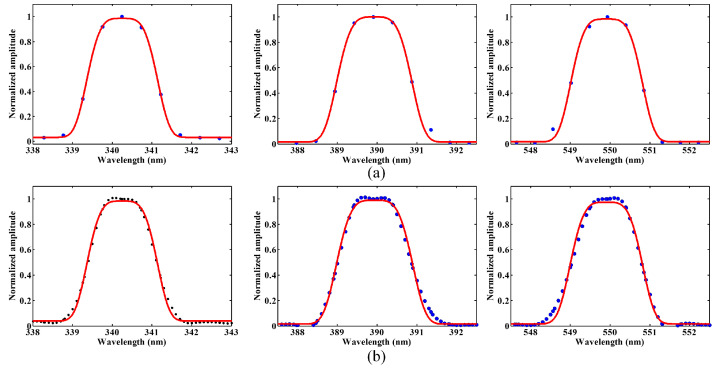
Blue dots are test data, red line is the fitted curve (**a**) The SRF of different wavelengths with a fixed echelle grating angle of the SFI and (**b**) the SRF of one pixel with many angles.

The SRF for the AAS was obtained using the extracted profile in Figure 7b, with the SFI grating located at a fixed angle. To improve the sampling frequency, the positions of the peaks within the equipment shift along a wavelength dimension by turning the echelle grating. The fixed pixel’s response to an echelle peak signifies the SRF of the relevant pixel. By implementing this procedure, the shape of the SRF is assessed with more sampling than is obtained by employing a spectral line. For the AAS, a super-Gaussian profile was utilized, which is characteristic of the slit homogenizer. Equation (4) represents the super-Gaussian profile.

(4) $$y=A_{2}+A_{1}\cdot e^{-{\left((\lambda -\lambda_{0})./c_{0}\right)}^{4})}$$

where *A*_1_, *A*_2_, *λ*_0_, *c*_0_ represent the fitting parameters. Figure 8a depicts a plot of the SRF sampling when recording the spectral line response with a fixed angle, while Figure 8b displays the enhanced sampling of the SRF with many angles. A functional fit relative to the data points of the plot is also provided in Figure 8b. Theoretically, the SRFs of the two methods should be consistent, and the consistency of the measured results of the two methods verifies the accuracy of the calibration results, utilizing denser sampling points that can characterize the SRFs with higher accuracy. As shown in Figure 8, the spectral resolution/sampling ratio of the AAS was typically four. Whereas for the dedicated slit function instrument, an improved sampling of the spectral resolution was achieved at least ten times. The use of SFI to obtain a higher sampling frequency resulted in a more accurately obtained spectral response function of the AAS. The SRF’s shapes from the Earth port were similar to a Gaussian curve with a flat top, with an FWHM of 1.728–1.82 nm for the AAS. The results showed a correlation of about 0.99, which indicates that the fitting function could describe the SRF of the instrument. For the short-wave ultraviolet wavelength, the silt function instrument has a lower SNR and will affect the accuracy of the measurement, but for the AAS, the measurement band of 340–550 nm has enough energy, which is the ideal test method.

**Figure 9:**
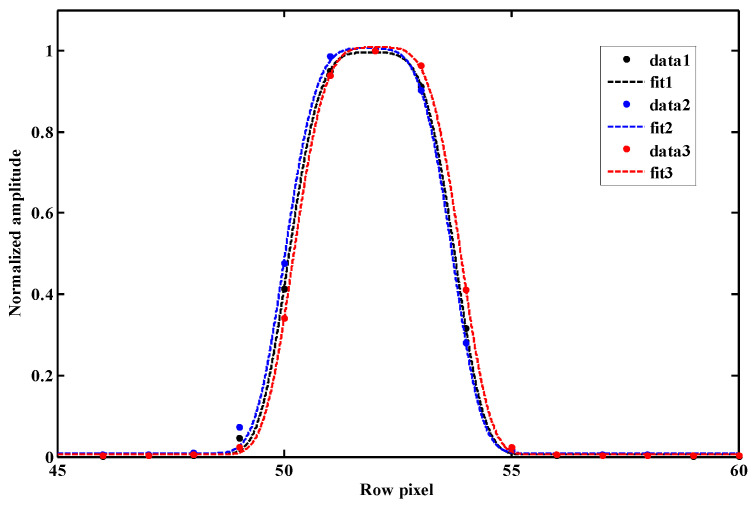
The SRF depends on three different temperatures.

The SRF’s shapes dependent on temperature were measured, as shown in Figure 9. The dots represent the measured data, the dotted line is a fitted curve, and distinct colors represent different temperatures (data1, data2, and data3 correspond to 19.9 °C, 14.1 °C, and 24.6 °C, respectively). The center of the curve varied slightly with temperature, as shown, which is consistent with Figure 6. In addition, when compared to the results at 19.9 °C, the FWHM under other temperatures shows a slight broadening, with 0.06 pixels.

### 3.3. Spectral Smile

**Figure 10:**
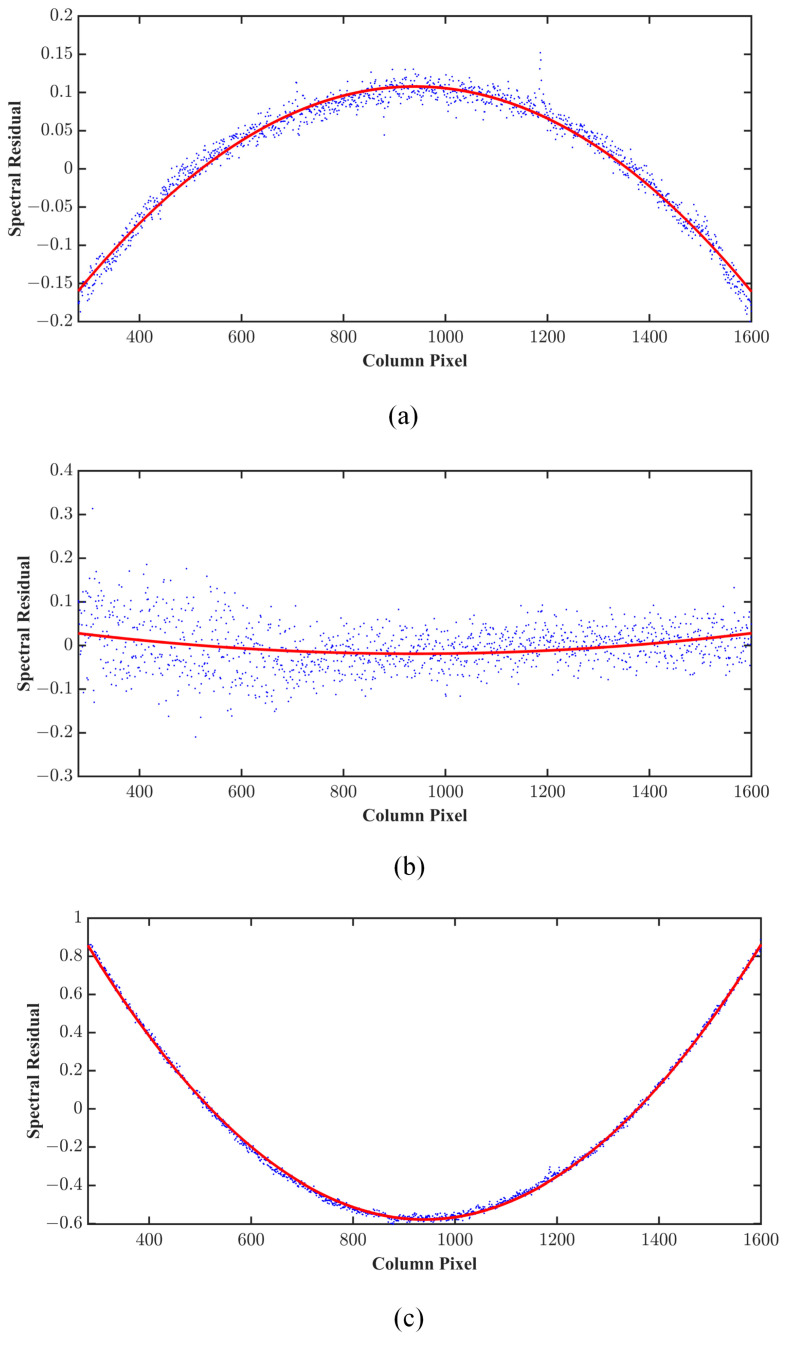
The spectral smiles at (**a**) 340 nm, (**b**) 400 nm, and (**c**) 550 nm. Blue dots are test data, red line is the fitted curve.

Setting the SFI at an angle, the spectrum can be scanned on the spatial array of the detector using a rotating platform, and the coordinates of all the spectra in the FOV of the AAS can be obtained. By fitting the spectral row number, which is dependent on the spatial column number, a straight line can be obtained. This straight line may tilt on the image surface. To correct the image tilt, the residual value of the fitted straight line is used, and then the residual and the spatial column are employed to fit the curve. By obtaining the highest and lowest points of the curve, the maximum space curvature can be obtained. Figure 10 shows the smile effect of differing spectra. The points denote the measurement data in Figure 10. Also, the solid line designates the fitted curve. The maximum smile represented 1.4 pixels at a wavelength of 550 nm. For the 340 nm smile, it was 0.27 pixels. The minimum denoted 0.2 pixels for 400 nm.

## 4. Conclusions

This research described the instrument’s characteristics and performance, alongside the pre-launch on-ground spectral calibration of an AAS. The data relating to the calibration process was obtained by illumination of the entrance port of the AAS via a vacuum chamber with dedicated on-ground testing equipment. For the pre-launch spectral calibration, all the spectral pixels were tested and determined from the tunable SFI fine-step scans. The wavelength ranged 338–551 nm with 1.728–1.82 nm spectral resolution. After running spectral calibration by controlling the different temperatures, the spectrum drift was measured. As the temperature changed from 14.1 to 24.6 °C, the center of the spectrum line had almost no change, but the FWHM varied by 0.06 pixels. The spectral smile was measured using the SFI, with a maximum of 1.4 pixels at 550 nm.

## Figures and Tables

**Table 1:** The GF-5B/AAS’s primary optical properties.

Parameters	Characteristics
Spectral Range (nm)	340–550
Spectral Resolution (nm)	<2.0 nm
FOV	±57°
Spatial Resolution	4 × 4 km (at nadir)
SNR	>1000 (10.89 μW/cm^2^. sr. nm)
Accuracy of Spectral Calibration	0.1 nm

**Table 2:** The properties of the spectral line source.

The Wavelength Calibration System	
Spectral Line Source (Hg) Wavelengths	365.016 nm, 404.657 nm, 435.834 nm, 546.075 nm
Spectral Line Source (Ar) Wavelengths	335.853 nm, 355.43 nm, 394.901 nm, 415.86 nm, 496.508 nm
Spectral Line Source (Kr) Wavelengths	377.342 nm, 450.235 nm, 473.900 nm, 476.574 nm
Diameter of Beam	15 mm
Divergence Angle	0.025°

**Table 3:** The properties of the slit function instrument.

SFI	
Irradiance at 50 cm	5.06 μW/cm^2^·nm@440 nm
Spectral Resolution	0.03 nm–0.06
Wavelength Range	250–1100 nm
Diameter of Beam	50 mm
Divergence Angle	0.05°
Raster Scribing	79.01 grooves/mm

## Data Availability

The data presented in this study are available upon request from the author.
